# On-surface synthesis of aligned functional nanoribbons monitored by scanning tunnelling microscopy and vibrational spectroscopy

**DOI:** 10.1038/ncomms14735

**Published:** 2017-04-03

**Authors:** Nataliya Kalashnyk, Kawtar Mouhat, Jihun Oh, Jaehoon Jung, Yangchun Xie, Eric Salomon, Thierry Angot, Frédéric Dumur, Didier Gigmes, Sylvain Clair

**Affiliations:** 1 Aix Marseille Univ, University Toulon, CNRS, IM2NP, Marseille 13397, France; 2 Aix Marseille Univ, CNRS, ICR, Marseille 13397, France; 3 Department of Chemistry, University of Ulsan, Ulsan 680-749, Republic of Korea; 4 Aix Marseille Univ, CNRS, PIIM, Marseille 13397, France

## Abstract

In the blooming field of on-surface synthesis, molecular building blocks are designed to self-assemble and covalently couple directly on a well-defined surface, thus allowing the exploration of unusual reaction pathways and the production of specific compounds in mild conditions. Here we report on the creation of functionalized organic nanoribbons on the Ag(110) surface. C–H bond activation and homo-coupling of the precursors is achieved upon thermal activation. The anisotropic substrate acts as an efficient template fostering the alignment of the nanoribbons, up to the full monolayer regime. The length of the nanoribbons can be sequentially increased by controlling the annealing temperature, from dimers to a maximum length of about 10 nm, limited by epitaxial stress. The different structures are characterized by room-temperature scanning tunnelling microscopy. Distinct signatures of the covalent coupling are measured with high-resolution electron energy loss spectroscopy, as supported by density functional theory calculations.

## Abstract (web summary)

On-surface synthesis, in which molecular units assemble and couple on a defined surface, can access rare reaction pathways and products. Here, the authors synthesize functionalized organic nanoribbons on the Ag(110) surface, and monitor the evolution of the covalent reactions by an unorthodox vibrational spectroscopy approach.

The emerging field of on-surface synthesis aims at producing novel and original reactions templated on the surface of a well-defined metal substrate^1^,^2^,^3^,^4^,^5^,^6^,^7^,^8^,^9^. The latter can be catalytically active and compels the confinement of the molecular precursors upon adsorption, allowing for the exploration of new reaction pathways in soft conditions. Robust covalent nanostructures can thus form directly on surfaces by combining supramolecular self-assembly processes with chemical activation. After exfoliation process, integration of the organic structures into electronic devices is envisioned, whereby exquisite and steerable properties are expected for a wide range of applications^10^,^11^,^12^. The on-surface synthesis strategy can lead, for example, to soft linear polymerization of alkyl chains^13^,^14^ or to the formation of well-defined extended nanoribbons^15^,^16^,^17^. A large variety of reactions has been explored to activate the formation of covalent bonds between instructed molecular building-blocks. Nevertheless, the community is still looking for a robust rationale before being able to effectively control and further develop this technological field. In fact, despite nearly a decade of successful demonstrations of on-surface covalent coupling, the choice and availability of efficient systems is still limited. For example, Ullmann coupling on surfaces is a very popular C–C coupling technique^3^,^18^,^19^ but has important drawbacks: atomic halogens are produced as byproducts impeding the coupling process^20^, and the high reactivity of the radical intermediates leads to the competitive formation of organometallics^21^,^22^. More recently, coupling of alkyne moieties has been demonstrated, but because it is non-specific, the on-surface reaction leads to the concomitant formation of a variety of chemical species^23^. Also, introducing specific functionalization into the as-formed covalent nanostructures while preserving the reactivity of the precursors is not straightforward^24^,^25^,^26^,^27^. Finally, and most importantly, non-ambiguous demonstration of the covalent nature of the structures is always challenging^21^,^28^,^29^,^30^. This is usually done through direct observation and measurement of the spatial extension by scanning tunnelling microscopy (STM)^21^ or by high-resolution atomic force microscopy (AFM) imaging^31^. Chemical characterization can be fulfilled using scanning tunnelling spectroscopy (STS)^28^,^32^ or photoemission spectroscopy (XPS/UPS)^33^,^34^,^35^,^36^,^37^ but the correct interpretation of the experimental data normally requires the development of advanced and extensive theoretical modelling.

High-resolution electron energy loss spectroscopy (HREELS) is one of the most advanced spectroscopy technique mainly used to obtain the vibrational signature of surface and adsorbate species. By assigning the vibrational modes, it is possible to evidence the chemical bonding between surface and adsorbate, between several adsorbates, or any chemical modification upon stimulation^38^,^39^,^40^,^41^.

The polycondensation of small aromatics containing oxygen is an appealing class of reactions for the creation of extended on-surface networks as it releases only harmless byproducts upon C–C coupling but it has been only scarcely and recently reported: the cyclotrimerization of acetyls^42^, the aldol condensation^43^, the reductive coupling of aldehydes^44^, and the *ortho* C–H functionalization of phenol derivatives^45^. The molecule *s*-indacene-1,3,5,7(2*H*,6*H*)-tetrone (also named ‘*Janus* dione'^46^ or in the following work, INDO_4_) belongs to the family of indanone derivatives. These compounds have been extensively studied in the literature, especially for the design of highly soluble planar heptacyclic polyarene structures (10,15-dihydro-5*H*-diindeno[1,2-*a*;1′,2′-*c*]fluorene), more commonly known as truxenes, that result from the trimerization of three indan-1-one units^47^. By increasing the content of ketone units per trimerizable units, similarly trimerization of indanedione derivatives can furnish truxenone analogues with three keto groups at the inner positions^48^. While generating symmetrical units such as in the case of INDO_4_, bifunctional tetraketoindacenes open the way towards carbonyl-functionalized microporous ladder polymers^49^.

In this report we present a covalent coupling mechanism for the polycondensation of the simple INDO_4_ precursor to form 1D functionalized nanoribbons. Dihydrogen is formed as byproduct, and the ketone functions are retained into the structures, thus preserving their functionality. Remarkably, we show that the surface-supported covalent coupling can be characterized by vibrational spectroscopy. The use of the surface-sensitive HREELS is here demonstrated as a unique and efficient tool to monitor *in situ* the covalent character of surface-supported transformations, positioning it as a promising technique for the field of on-surface synthesis. Upon deposition of INDO_4_ on the well-defined Ag(110) surface, extended supramolecular networks are observed. The cohesion is ensured by a dense network of hydrogen bonds, thanks to the presence of peripheral ketone groups and hydrogen atoms. C–H bond activation and homo-coupling of the precursors is achieved by thermal activation. For an annealing temperature of 300 °C, the anisotropic substrate acts as an efficient template fostering the formation and the alignment of covalent nanoribbons along the [001] direction. The length of the ribbons is limited to two to three monomeric units and is gradually increasing with increasing annealing temperature. A supplemental coupling mechanism is activated at higher temperatures (≥400 °C) producing longer transverse nanoribbons. The different structures are characterized by room-temperature STM. Distinct signatures of the covalent coupling are measured with HREELS by observing the stretching mode of the as-formed C=C bond between precursors in addition to the appearance of an exalted macrocycle-breathing mode, as supported by density functional theory (DFT) calculations.

## Results

### Supramolecular phase

**Figure 1:**
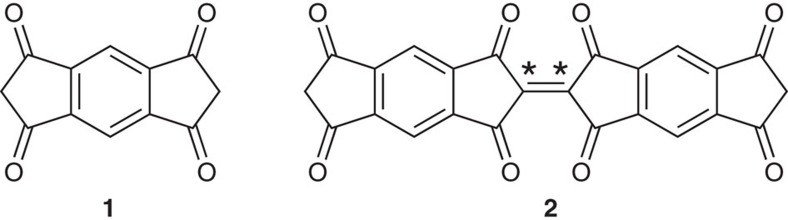
INDO_4_ molecule. Schematic representation of *s*-indacene-1,3,5,7(2*H*,6*H*)-tetrone (INDO_4_, **1**)^46^ and related dimer (**2**).

**Figure 2:**
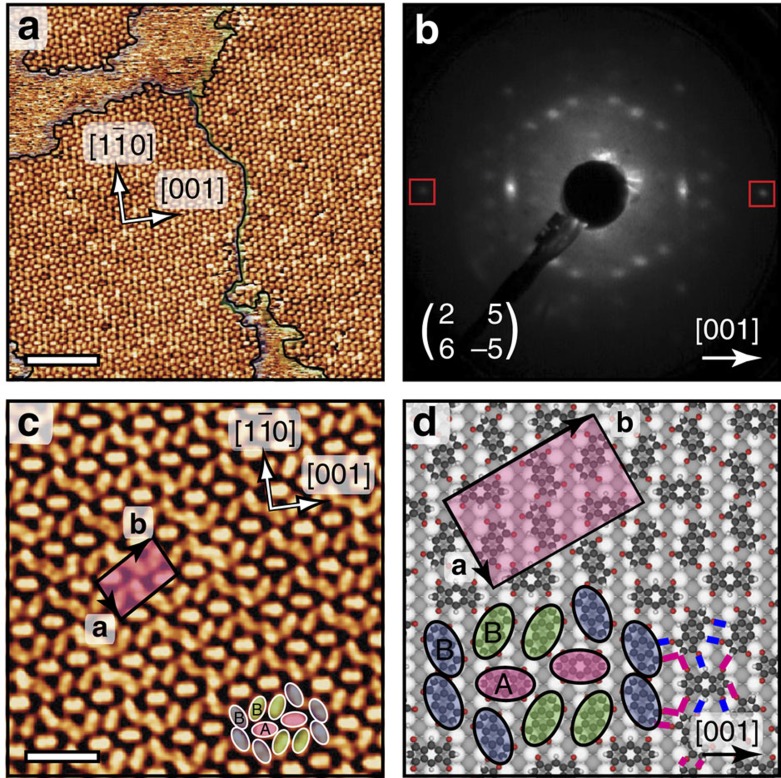
Supramolecular phase. Self-assembled structures formed by INDO_4_ upon adsorption on a Ag(110) surface held at 50 °C. (**a**) Large-scale scanning tunnelling microscopy (STM) image showing 2D molecular islands of opposite chirality highlighted by violet and green contours and (**b**) corresponding low-energy electron diffraction pattern (recorded at 17.3 eV) with Ag spots marked by the square outlines. (**c**) High-resolution STM image and (**d**) corresponding tentative model of the superstructure with rectangular unit cell and lattice directions indicated. Ag atoms in the topmost close-packed rows are shown white, while lower lying Ag atoms are grey. The formation of C=O···HC intermolecular hydrogen-bonding is indicated by blue and pink lines. Scale bars (**a**) 10 nm; (**c**) 3 nm.

The INDO_4_ molecule^46^ (**1**, Fig. 1) consists of a *s*-indacene core whereby each five-membered ring is composed of two carbonyl groups (at the 1,3,5,7-positions) and one CH_2_ (at the 2,6-positions). The sub-monolayer deposition of this molecule on Ag(110) surface (Supplementary Fig. 1) kept at room temperature leads to the formation of small domains poorly ordered (Supplementary Fig. 2) whereby the intermolecular cohesion is ensured by a dense network of hydrogen bonds^50^,^51^,^52^. The subsequent surface annealing or direct molecule deposition on a substrate held at a slightly elevated temperature (50 °C) fosters the growth of extended two-dimensional (2D) highly ordered molecular islands with two chiral domains, as shown in the large-scale STM image and corresponding low-energy electron diffraction (LEED) pattern of Fig. 2a,b, respectively (Supplementary Fig. 3). This close-packed structure appears as a complex pattern whereby two [001]-oriented molecules (type A, see pink ovals in Fig. 2c) are encapsulated in elongated 10-membered cavities (with type B molecules, see blue and green ovals in Fig. 2c). The unit cell of this molecular assembly comprises 6 molecules of three distinct orientations and corresponds to a commensurate (2 5; 6 -5) superstructure with respect to the Ag(110) surface. The tentative model of the tiling pattern as shown in Fig. 2d was derived from superimposing carefully scaled ball-and-stick models of INDO_4_ onto the experimental STM images, and subsequently placed on the space-filled model of the silver surface. This routine allows not only identifying the observed assembly and azimuthal orientations of the molecules, but also provides hints on their intended adsorption sites and intermolecular interactions. The model thus indicates an adsorption configuration of the type A molecules with their phenyl ring being most probably in the on-top site of the first Ag layer, and with their long axes being aligned with the [001] direction. In such configuration, all O atoms would be also lying close to on-top sites of Ag substrate, as it is also observed for other molecules^53^. The remaining type B molecules have two different symmetric orientations rotated by ±25° with respect to [1
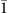
0] directions, see green and blue ovals in Fig. 2c,d.

Careful inspection of island perimeters and vacancy defects within this structure was performed to extract the most energetically favourable adsorption site. The molecular islands are preferentially terminated by type A [001]-oriented INDO_4_ molecules, while the defects in island interiors are mainly found for type B molecules (Supplementary Fig. 4). Therefore one can conclude that the [001]-oriented (type A) molecules are more stable with possibly stronger surface interaction. The presence of two distinct adsorption sites A and B in this assembly is additionally confirmed by the difference in molecular contrast. Specifically, the backbones of type A and type B molecules can be visualized in STM images as oval-shaped dim and bright protrusions, respectively, or occasionally in a reverse way due to the change of tip condition (see Supplementary Fig. 5). The distances of 2.6±0.1 and 2.7±0.2 Å between O and H atoms of neighbouring molecules derived from the tentative model in Fig. 2d indicate that the stability of such complex structure is further enhanced by the formation of C=O···HC intermolecular hydrogen bonds^54^,^55^.

**Figure 3:**
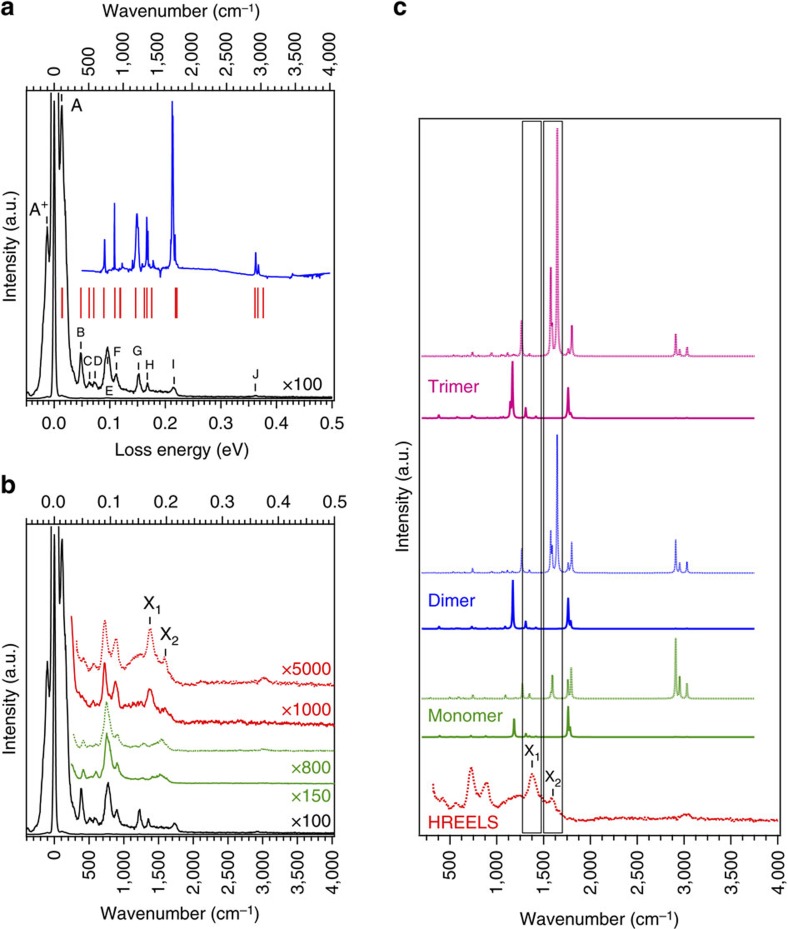
Vibrational characterization. (**a**) High-resolution electron energy loss (HREELS) spectrum of INDO_4_ thick film (black), together with infrared spectrum of the INDO_4_ powder (blue) and theoretical infrared active modes (red sticks). The corresponding vibrational modes are assigned in Table 1. (**b**) HREELS spectra of the INDO_4_: thick film (black), monolayer (supramolecular phase, green), monolayer annealed at 380 °C (polymeric phase, red). Full line curves were recorded in specular geometry while dashed lines were taken in off-specular geometry. All spectra were normalized to the intensity of peak E at 775 cm^−1^. (**c**) Theoretical spectra of the infrared (full line) and Raman (dotted line) active vibrational modes for the monomer, the dimer and the trimer together with the HREELS spectrum obtained after annealing at 380 °C. Theoretical data have been scaled to the infrared spectrum of the pristine INDO_4_. For the sake of comparison the calculated infrared active modes of the monomer, dimer and trimer have been normalized to the peak at 1,760 cm^−1^. A similar normalization was done for the Raman active modes using the peak at 1,790 cm^−1^.

Optical methods such as infrared and Raman spectroscopies are powerful techniques of probing the vibrational modes of atoms and molecules in bulk material. However, when employed to the study of adsorbates on surfaces, the signals are usually very weak and difficult to extract from the background. And this becomes even more difficult when the substrate is a metal because of its high reflectivity in the infrared range. Due to the use of suitably chosen low-energy electrons, HREELS is extremely surface-sensitive. Despite the fact that its resolution is substantially less than in optical spectroscopies, HREELS is an adequate and efficient tool to study the vibrational modes of adsorbed molecules on metals. Figure 3a exhibits the HREELS data recorded on the pristine INDO_4_, that is, the thick film of INDO_4_/Ag(110), and the infrared spectrum of the INDO_4_ powder, together with the positions of infrared active modes as calculated from DFT. The good overall agreement between the different experimental set of data and the theoretical one allowed us to assign the different loss structures observed, as reported in Table 1. Figure 3b displays HREELS data of the pristine INDO_4_ (thick film, black curve), which serves as a reference to assign the peaks, together with that of the monolayer regime (supramolecular phase, green curve). No essential difference is observed between the loss signature of the pristine INDO_4_ and that of the monolayer phase. Mainly, peaks G to I are no longer present, or at least of weaker intensity. To explain this issue one has first to notice that loss features G, H and I mostly correspond to in-plane vibrational modes, that is, parallel to the surface, and do not belong to a fully symmetric representation (cf. Table 1). Then we should consider that in specular geometry, and for highly ordered film, the dipole scattering is the dominant mechanism. In such conditions, the selection rules state that only vibrations that belong to the totally symmetric representations can be observed^38^. Therefore according to the dipole selection rules and due to the symmetry of the involved G, H and I features, they cannot be observed at monolayer completion and in specular geometry. The fact that there are no substantial modification of the vibrational structure upon adsorption at the monolayer regime further suggests that the interaction between the INDO_4_ molecules and the underlying Ag(110) substrate is weak, that is, van der Waals interactions. The C–H stretching mode for the monolayer regime is measured at an energy of 3,017 cm^−1^, in contrast to the thick film case where two components at 2,944 and 3,041 cm^−1^ can be observed, corresponding to the C–H *sp*^3^ and C–H *sp*^2^ stretching modes, respectively^38^ (Supplementary Fig. 6). This suggests that the molecules underwent a dehydrogenation reaction, at least partially, leading to the disappearance of all CH_2_ groups. Dehydrogenation is sometimes observed to occur on silver surfaces already at room temperature or upon mild annealing^45^,^56^. The C–H stretching mode is observed in off-specular geometry only, that is, when the dipolar scattering mechanism is not the dominating one, in agreement with the flat lying adsorption observed by STM.

### Polymeric phase

**Figure 4:**
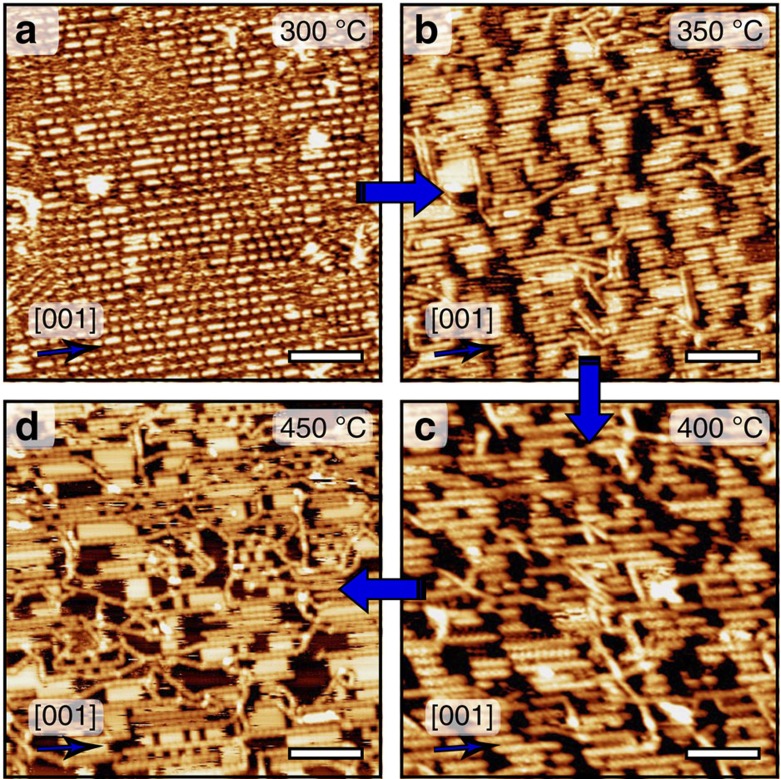
Nanoribbon formation and their evolution upon further annealing. Scanning tunnelling microscopy images of the on-surface synthesis of aligned functional nanoribbons by the subsequent surface annealing or the direct deposition of INDO_4_ on Ag(110) substrate held at (**a**) 300 °C (**b**) 350 °C (**c**) 400 °C and (**d**) 450 °C. Scale bars, 8 nm.

Covalent coupling is sequentially activated at more elevated temperatures (Fig. 4; Supplementary Fig. 7). Covalent dimerization (homo-coupling reaction) takes place through release of hydrogen and creation of a new C*=C* double bond (for example, dimer **2**, Fig. 1). Molecular dihydrogen byproduct is expected to spontaneously desorb from noble-metal substrates under the conditions employed^57^. We assume here that the possibility of a C–C single bond formation is not allowed because the oligomeric structures are imaged perfectly homogeneous and parallel to the surface plane (Supplementary Fig. 8). This hypothesis is also supported by previous works devoted to the dimerization of 1,3-indandione derivatives where the dimer 2,2′-biindanylidene-1,3,1′,3′-tetraone could be formed by homo-coupling of 2,2-dichloro-1,3-indandione in the presence of copper^58^. Similarly, the reported conversion of 3,3′-dihydroxy-2,2′-bi-1*H*-indene]-1,1′dione to 2,2′-biindanylidene-1,3,1′,3′-tetraone by oxidation with 2,3-dichloro-5,6-dicyano-1,4-benzoquinone (DDQ)^59^ is sustaining the possibility of an oxidation assisted by the metal surface providing the dimer **2** (Fig. 1).

**Figure 5:**
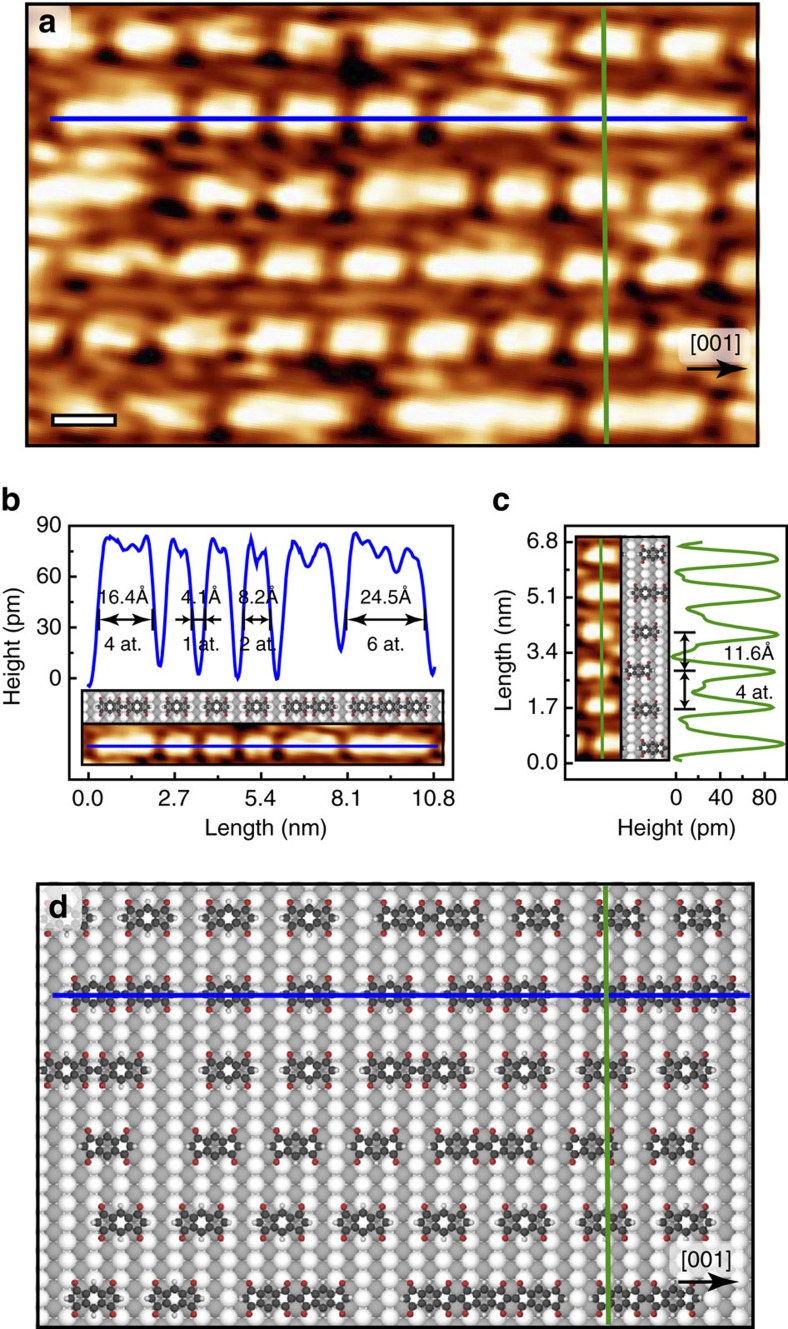
First polymerization steps. Deposition of INDO_4_ on a substrate kept at 300 °C lead to the formation of parallel rows composed of single isolated molecules, head-to-head covalently bonded dimers and trimers that are all oriented along [001] direction. (**a**) High-resolution scanning tunnelling microscopy (STM) image. Scale bar, 1 nm. (**b**) Line profiles along a single molecular row in the [001] direction and (**c**) across parallel rows in the transverse [1
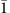
0] direction, indicated by blue and green lines in (**a**), respectively. 1 at. corresponds to 1 Ag lattice parameter. Epitaxy is preserved in both directions. (**d**) Tentative model of the molecular network presented in the STM image of (**a**).

Annealing of the supramolecular phase, or deposition of INDO_4_ on a substrate held at 300 °C results in the formation of parallel molecular rows running along the [001] direction (Figs 4a and 5a). Within each row, elongated features of different sizes can be distinguished. Their lengths are restricted to 8.2, 16.4, 24.5 Å, consistent with the sizes of single isolated molecules, head-to-head covalently bonded dimers and trimers, respectively. Also, the distance of 4.1 Å between individual units along [001]-oriented rows (Fig. 5b) corresponds nicely to a single atomic periodicity of the underlying Ag surface, thus entailing molecular chain growth in perfect registry with the substrate. It is expected that INDO_4_ and its polymerized forms preferentially adopt type A adsorption site, which corresponds to the most stable configuration found in the room temperature supramolecular phase of Fig. 2, or eventually a shifted site by sliding by half a unit cell in the [001] direction. Remarkably, in all cases O atoms are similarly lying close to on-top sites of the Ag substrate (Fig. 5d). Besides, the strictly uniform inter-row spacing of 11.6 Å in the [1
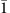
0] direction corresponds to four-atomic distance of the Ag(110) substrate (Fig. 5c; Supplementary Fig. 9a,b).

At the temperature of 300 °C the polymerization process is restricted to its first steps, providing exclusively dimers or trimers. Higher activation energy is required to extend the reaction to longer oligomers. In fact, the coupling mechanism for a dimer or a trimer may be significantly different than for an isolated molecule because fine epitaxial effects can take place due to the exceptionally strong catalytic implication of the metal substrate^19^,^45^,^60^. Also, the diffusivity of dimers and trimers is probably reduced, lowering similarly the reaction probability.

**Figure 6:**
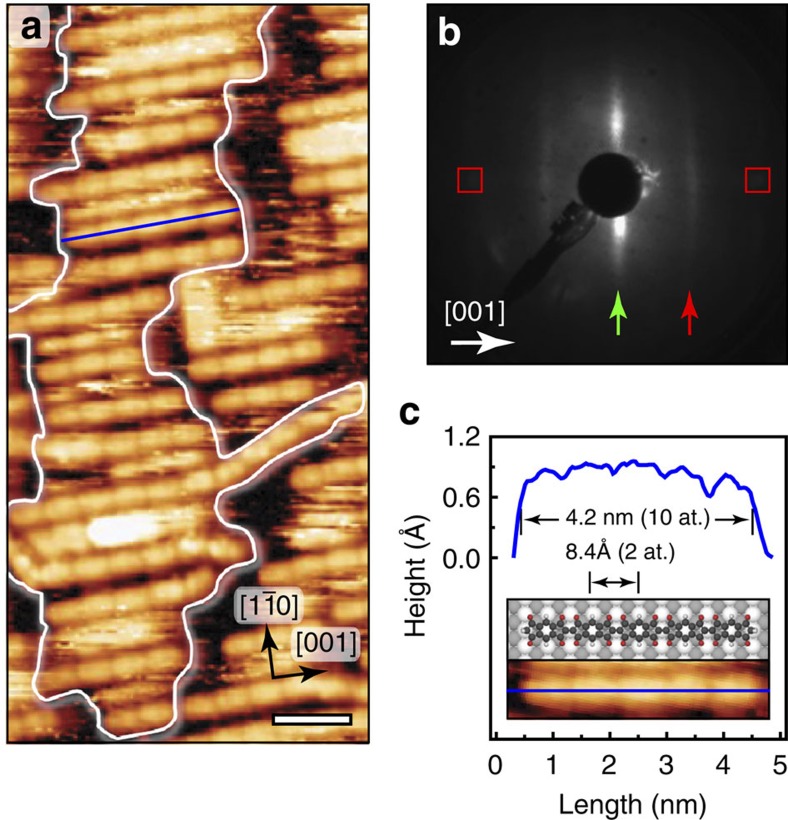
Growth of functional nanoribbons upon surface annealing to 350 °C. (**a**) High-resolution scanning tunnelling microscopy image showing head-to-head covalently bonded INDO_4_ chains. Scale bar, 2 nm. (**b**) Corresponding low-energy electron diffraction pattern (recorded at 23.3 eV) with Ag spots marked by the square outlines. The presence of vertical stripes, extended along [1
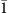
0], indicates non-uniform inter-chain spacing along the [1
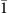
0] direction (green arrow) and molecular periodicity within polymeric chains oriented along [001] direction (red arrow). (**c**) Line profile along a pentameric molecular chain oriented in the [001] direction, indicated by blue line.

Annealing of the surface to 350 °C produces further modification of the assembly, namely the growth of longer straight covalent chains, with lengths corresponding principally from dimers to hexamers, still running along the [001] direction (Figs 4b and 6a). The monomeric units are distinguishable inside the covalently bonded chains with a periodicity of 8.2 Å (Fig. 6a,c). The ends of the parallel chains interact with each other and tend to accommodate in the perpendicular direction, leading to the formation of domains drastically elongated along the [1
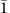
0] direction, as highlighted by a white line in Fig. 6a. The chain interspacing is here non-uniform but corresponds mainly to integer numbers of substrate Ag atomic rows, providing thus a non-perfectly ordered structure in the [1
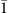
0] direction. The corresponding LEED pattern confirms this poor order in the form of elongated spots along the [1
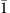
0] direction, in agreement with the Fast Fourier transform (FFT) of the STM images (Supplementary Fig. 9c,d). The periodic sequence generated inside the polymeric chains is represented as dim stripes along the [001] direction in the LEED pattern (Fig. 6b). The reciprocal distance corresponds to ∼8.2 Å (2 at.) or twice the substrate atomic periodicity in this direction, confirming the epitaxial growth of the polymeric chains. Occasionally, few covalent chains, referred to as transverse, are seen by STM in-between the patches, sometimes not strictly straight and occasionally aligned with [1
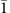
0] direction. Also, a few unreacted molecules are still present on the surface.

The reason for the limited size of the chains along [001] may arise from epitaxial stress. DFT calculations of free-standing oligomers (up to the pentamer) suggest a good epitaxy with the substrate: the length increase of the oligomer by adding one monomeric unit is 8.3±0.1 Å according to DFT while the size of 2 unit cells of Ag substrate along [001] is 8.2 Å. The small discrepancy (0.1 Å per monomer) is probably responsible for the existence of a chain size limit (hexamer to octamer, 5 to 7 nm) above which epitaxy is prohibited. Longer chain can grow only by deviating from perfect [001] epitaxial alignment. This further suggests that the reaction mechanism is intimately related to the adsorption configuration of the reactants, as it was shown for other on-surface syntheses^19^,^60^.

**Figure 7:**
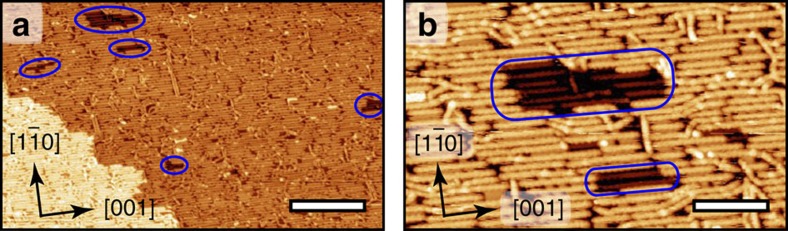
Full monolayer of aligned nanoribbons formed by INDO_4_ deposition on Ag(110) surface kept at 350 °C. (**a**) Large scale and (**b**) high-resolution scanning tunnelling microscopy images. Pit formation is highlighted by blue oval contours. Scale bar, (**a**) 30 nm; (**b**) 10 nm.

Remarkably here the coverage of the surface can be increased to monolayer range, thus providing a complete sheet of well-aligned organic nanoribbons (Fig. 7). Although it is suggested that at room temperature the molecules are weakly interacting with the substrate, the situation is very different at annealing temperature along the reaction pathway. Indeed, for high coverage and after annealing at 350 °C small pits of a few nm wide are sparsely observed inside the Ag substrate (Fig. 7b). The extraction of Ag adatoms from the surface is thus probably required in the reaction mechanism. Vacancy pits on Ag(110) are expected to annihilate readily at elevated temperature^61^. The molecular layer is thus also stabilizing, at least partially, their formation upon cooling back to room temperature. Indeed, small molecular structures are usually found inside the pits. Several STM images were sequentially recorded at room temperature to confirm the stability of covalent chains and vacancy pits, and the presence of isolated molecules (Supplementary Fig. 10; Supplementary Movie 1). The images taken on the same location demonstrate the presence of very mobile isolated molecules located in between the chains and in the pits of the surface. These molecules appear as structureless bright features, with slightly higher apparent height. On the other hand, the polymeric chains are perfectly stable (not diffusing) in this assembly within the measurement timeframe (≥1 h).

At 400 °C annealing temperature, the molecular structure (Fig. 4c) is similar to that at 350 °C with no substantial elongation of the [001]-aligned polymeric chains. HREELS data for the polymer phase annealed at 380 °C are shown in Fig. 3b (red curves). Two clear peaks, labelled X_1_ and X_2_ appeared. As reported in Table 1, peak X_1_ can be assigned to an infrared active macrocycle-breathing mode and/or an in-plane bending mode of the *sp*^2^ C–H bonds (Supplementary Movies 2,3). According to DFT calculations, both modes are already observable for the monomer case but their intensity increases with the chain length (for dimers and trimers, see Fig. 3c). Therefore, since the HREELS signature of these features is much more intense upon annealing, one could emphasize, in relation with the STM data, that the homo-coupling reaction effectively occurs upon annealing. But more stimulating is the peak X_2_. According to its frequency this peak can only be assigned to two different, fully symmetric Raman active modes corresponding to a macrocycle-breathing mode and, most interestingly, to a C*=C* stretching mode (Table 1; Supplementary Movies 4,5). According to DFT calculations, the former mode is already observable for the monomer but its intensity is significantly increased for dimers and trimers (Fig. 3c). Also, the latter mode only appears upon annealing and oligomerization. In fact, peak X_2_ has a width larger than the elastic peak and can thus include in principle these two close but hardly distinguishable contributions (Supplementary Fig. 11). The presence of the peak X_1_ and the broader peak X_2_ thus clearly highlights the fact that annealing induces an on-surface reaction towards an effective homo-coupling of the INDO_4_ molecules. The signature of the C–H stretching mode (peak J) did not evolve upon annealing (Supplementary Fig. 6). A single component corresponding to C–H *sp*^2^ stretching mode is measured in off-specular geometry only. It is thus suggested that the dehydrogenation reaction, that occurred already in the supramolecular phase and that led to the disappearance of all CH_2_ groups, represents a first intermediate state towards the polymerization reaction.

**Figure 8:**
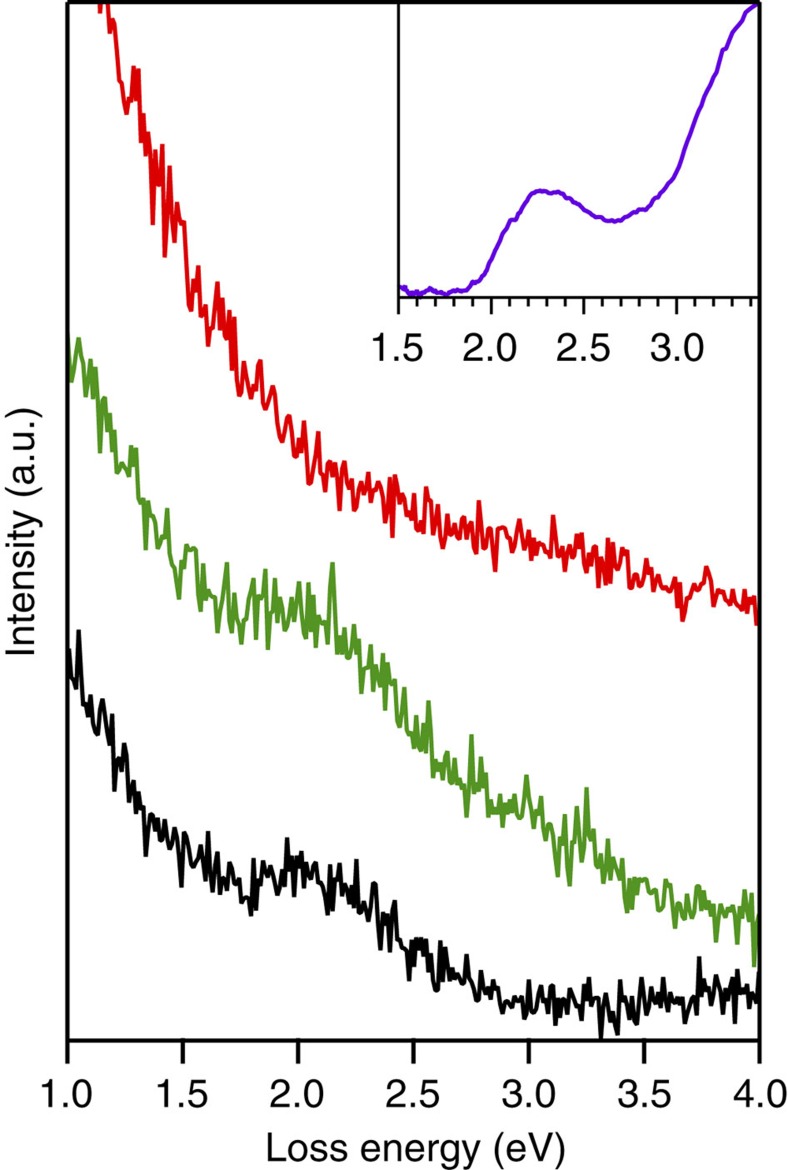
High-energy loss region. High-resolution electron energy loss (HREELS) spectra from 1 to 4 eV of the INDO_4_: thick film (black), monolayer (green), monolayer annealed at 380 °C (red). Top-right inset ultraviolet–visible spectrum of pristine INDO_4_ molecule in acetone.

To further reinforce the conclusions drawn from the STM data and the analysis of the vibrational modes of the system, we studied the evolution of the electronic signature of the system with HREELS by analysing high-energy losses, that is, above 1 eV. For organic conjugated materials, this region corresponds to the excitation of the lowest energetic electronic transitions, mainly between the Highest Occupied Molecular Orbital (HOMO) of the molecules and the Lowest Unoccupied Molecular Orbital (LUMO). Figure 8 displays HREELS spectra of the thick film (black), monolayer (green) and monolayer annealed at 380 °C (red), together with ultraviolet–visible data from the pristine molecules. In the case of the thick film there is a large loss structure at about 2.1 eV (∼0.5 eV wide). This structure matches the one observed by ultraviolet–visible spectroscopy and is attributed to the lowest electronic transition between the HOMO and the LUMO of the pristine INDO_4_. At monolayer coverage and prior annealing, this loss feature is still present demonstrating that the electronic structure of INDO_4_ is more or less unchanged upon adsorption, as expected considering the weak molecule-substrate interaction^62^,^63^. After annealing, the loss structure at 2.1 eV is no longer distinguishable. There might be two main reasons for such observation. First, upon annealing some molecules would have desorbed and the HREELS signal became too weak. Second, due to the polymerization there is a bathochromic effect and the feature of the peak is hidden in the HREELS background which is higher at lower loss energy. According to the *in situ* STM measurements performed after annealing but prior to HREELS, the INDO_4_ coverage was still high, estimated to be 0.8 Ml (Supplementary Fig. 12). Therefore we believe that the fade of the loss structure at 2.1 eV confirms the polymerization upon annealing. Indeed, the decrease of the HOMO-LUMO gap is characteristic of the oligomerization of *π*-conjugated 1D or 2D organic systems^37^,^64^,^65^,^66^. Accordingly, the lowest excitation energies mainly from HOMO to LUMO evaluated by means of the time-dependent DFT (TD-DFT) method are 3.36, 1.98, 1.95, 1.93, and 1.92 eV for the monomer, dimer, trimer, tetramer and pentamer, respectively (see Supplementary Table 1 for a comparison of different functionals). Although our approach does not take into account the influence of the substrate interaction and electronic screening effect^67^,^68^, and intermolecular interactions, such as *π*–*π* stacking and hydrogen bonding, the computational results clearly demonstrate that a drastic red-shift of the transition peak appears at the initial step of oligomerization, that is, the formation of dimers, and then the lowest excitation energy is gradually reduced as the length of molecular ribbon increases.

At 450 °C annealing temperature the formation of transverse chains is significantly increased (Fig. 4d). These covalently bonded transverse chains thus likely originate from a different and subsequent reaction pathway, whereby the epitaxial conditions are lost. In fact most transverse ribbons are seen as continuous extensions of the finite size [001]-aligned chains. The deviation from the [001] orientation allows for relaxing the epitaxial stress that limits the primary chain growth. At higher temperatures additional conformational configurations can be extensively explored, allowing for the activation of a supplemental reaction pathway for non-aligned monomers. Additional chain growth is possible because isolated and unreacted molecules are still available at high temperature, as mentioned above for the 350 °C experiment. Finally, at 500 °C, the structure does not show significant modification with respect to that obtained at 450 °C (Supplementary Fig. 13). The observation of preserved covalent nanoribbons after such high annealing temperature attests for their remarkably high robustness.

In this work, we introduced a chemical functionality to perform surface-supported C–H bond activation and homo-coupling reaction. The combination of STM imaging and HREEL spectroscopy proved very efficient to effectively characterize the chemical transformations. The presence of reactive functional groups (carbonyl oxygen) preserved along the reaction path greatly enhanced the epitaxy of the precursors, which most probably facilitated the coupling mechanism. Extended theoretical support would now be required to elucidate the complex mechanism usually encountered in such surface-supported C–C coupling^69^. In particular, the role of the prior hydrogen-bonded self-assembly is probably essential to the formation of the first dimerization steps. In fact the original precursor presented here is highly versatile and can produce a large variety of supramolecular or covalent structures on other coinage metal surfaces, as we will show in future publications. Our results confirm that the characterization tools employed in the blooming field of on-surface synthesis and the advanced description of such well-defined systems are able to provide an exquisite understanding of the fine physicochemical processes taking place.

## Methods

### Sample preparation

The experiments were performed in two distinct ultra-high vacuum systems working at base pressures in the low 10^−10^ mbar range and equipped with LEED and standard facilities for sample preparation. The first experimental set-up was used for the STM measurements while the second set-up was dedicated to combined STM/HREELS studies.

The single-crystal Ag(110) surface was cleaned by several cycles of Ar^+^ bombardment followed by annealing. INDO_4_ molecules were provided by Sigma-Aldrich as rare and unique chemical. The precursors were thoroughly degassed prior to deposition onto the atomically clean substrate held at different temperature (room temperature to 400 °C). No difference could be observed for the structures formed by room temperature deposition and subsequent annealing or by deposition on a hot substrate. The molecular powder was thermally sublimated from an evaporator heated to 150 °C for typical dosing time of 30 min to allow tuning the formation of well-ordered supramolecular or covalently bonded organic films.

### STM measurements

The self-assemblies of as-deposited INDO_4_ supramolecular architecture and the molecular film modified by thermal treatment were studied with a commercial Omicron VT-STM system operated at room temperature. The STM images were acquired in constant current mode with typical tunnelling current *I*_T_≈0.3 nA and sample bias *V*_T_≈1–1.5 V. All images were subsequently calibrated using atomically resolved images of the well-known Ag(110) surface. Images were partly treated with the free software WSXM^70^. The STM images allow to unambiguously identify organization and orientation of single molecules in H-bonded networks, and to resolve important aspects of internal structure within covalent chains.

### HREELS measurements

HREELS measurements were carried out using a VSI DELTA 0.5 spectrometer. All spectra were recorded with the same incident electron beam energy of 5.8 eV, measured from the cutoff of the loss spectra, and with a typical energetic resolution of about 5 meV, estimated from the full width at half maximum of the elastic peak. Scattering geometry: *θ*_i_=*θ*_s_=64° for specular geometry, *θ*_i_=64°, *θ*_s_=58° for off-specular. For the high-loss measurements in off-specular geometry: *θ*_i_=64°, *θ*_s_=48°.

### UV/Vis measurements

Ultraviolet–visible data were carried out in acetone and measured using a double-beam spectrophotometer SAFAS DES 190, in the range from 350 to 900 nm with a typical resolution of 4 nm.

### Infrared measurements

Infrared measurements were recorded from 400 to 4,000 cm^−1^ using a Bruker IFS 66/S Fourier transform infrared spectrometer equipped with a HgCdTe detector. The data were recorded on INDO_4_ powder, in attenuated total reflection (ATR) mode, using a Ge module and a typical resolution of 1 cm^−1^.

### DFT calculations

DFT calculations at the gas phase were carried out to support experimental data, where the geometric and vibrational structures of monomer and all oligomers (from dimer to pentamer) were evaluated using M06-2X hybrid functional^71^ and 6-311++G(2d,p) basis set implemented in Gaussian 09 software package^72^. To compare the vibrational frequencies obtained from DFT calculations with experimental values, we scaled the computational data to the experimental infrared data using a compression factor of 0.929 and a rigid shift of 35 cm^−1^. In addition, the lowest excitation energy was also evaluated using the time-dependent DFT (TD-DFT) method at the same level of theory. We used the ultrafine grid in numerical integration and the tight self-consistent field convergence criterion in all calculations.

### Data availability

The data that support the findings of this study are available from the corresponding author upon reasonable request.

## 

## Additional information

**How to cite this article:** Kalashnyk, N. *et al*. On-surface synthesis of aligned functional nanoribbons monitored by scanning tunnelling microscopy and vibrational spectroscopy. *Nat. Commun.* **8,** 14735 doi: 10.1038/ncomms14735 (2017).

**Publisher's note**: Springer Nature remains neutral with regard to jurisdictional claims in published maps and institutional affiliations.

## Supplementary Material

Supplementary Information Supplementary Figures and Supplementary Table.

Supplementary Movie 1 STM movie, 45 × 52 nm^2^, corresponding to a total real time of 52 min

Supplementary Movie 2 Vibrational mode assigned to peak X1a

Supplementary Movie 3 Vibrational mode assigned to peak X1b

Supplementary Movie 4 Vibrational mode assigned to peak X2a

Supplementary Movie 5 Vibrational mode assigned to peak X2b

## Figures and Tables

**Table 1:** Vibrational modes assigned to HREEL spectra.

	**Label**	**HREELS**	**Infrared**	**Theory IR act. (sym.)**	**Theory R act. (sym.)**	**Assignment**
TF	A+	−107	—	—	—	CCCH_2_ OPB*
	A	107	—	112 (*B*_1*U*_)	—	CCCH_2_ OPB
	B	387	—	387 (*B*_3*U*_)	—	CCCO IPB
	C	513	—	507 (*B*_1*U*_)	498 (*A*_*G*_)	CH OPB
	D	593	—	575 (*B*_3*U*_)	565 (*B*_3*G*_), 589 (*A*_*G*_)	macro. OPB
	E	775	732	721 (*B*_3*U*_)	742 (*A*_*G*_)	macro. br., CC str.
	F	903	875	880 (*B*_1*U*_)	—	CH OPB
	G	1225	1201	1182 (*B*_3*U*_)	1197 (*B*_1*G*_)	macro. br., CH *sp*^2^ IPB, wag. CH *sp*^3^
	H	1350	1344, 1361	1346 (*B*_3*U*_)	1346 (*A*_*G*_)	sci. CH *sp*^3^
	I	1740	1713, 1723	1760 (*B*_2*U*_), 1781 (*B*_3*U*_)	1758 (*B*_1*G*_), 1793 (*A*_*G*_)	CO str.
	J	2944, 3041	2925, 2963	—	2911 (*A*_*G*_), 2952 (*B*_2*G*_), 3030 (*A*_*G*_)	CH str. *sp*^3^, CH str. *sp*^2^
Annealed ML	X_1_	1383	—	1307 (*A*_*U*_)	—	macro. br.
			—	1416 (*A*_*U*_)	—	macro. br., CH *sp*^2^ IPB
	X_2_	1600	—	—	1574 (*A*_*G*_)	macro. br., C*=C* str.
			—	—	1644 (*A*_*G*_)	C*=C* str.

br., breathing mode; IPB, In-Plane Bending; IR act., Infrared active mode; macro., macrocycle; ML, monolayer; OPB, Out of Plane Bending; R act., Raman active mode; sci., scissor mode; str., stretching mode; TF, thick film; wag., wagging mode.

Values are given in cm^−1^.

^+^Indicates a gain process, the counter part of the loss one.
