# Supplementary material for: On-surface synthesis of aligned functional nanoribbons monitored by scanning tunnelling microscopy and vibrational spectroscopy
Source: Nat Commun. 2017 Apr 3;8:14735. doi: 10.1038/ncomms14735 (PMC5382267; doi:10.1038/ncomms14735)
Supplement: Supplementary Information — Supplementary Figures and Supplementary Table. [file ncomms14735-s1.pdf]

Supporting Figures

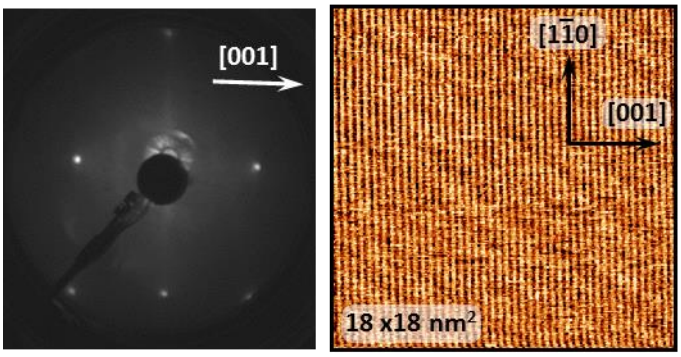

Supplementary Figure 1. LEED pattern of the clean Ag(110) and corresponding STM image exhibiting atomic resolution. This data allowed for the correct assignment of the orientation in the STM images.

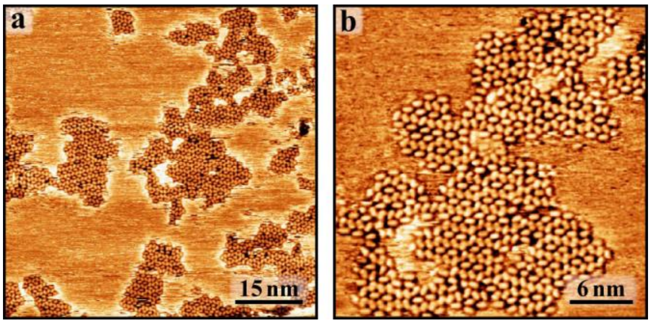

Supplementary Figure 2. Sub-monolayer deposition of $INDO_4$ on Ag(110) surface kept at room temperature: (a) large-scale STM image and (b) close-up view of small poorly ordered molecular domains stabilized by a dense network of hydrogen bonds.

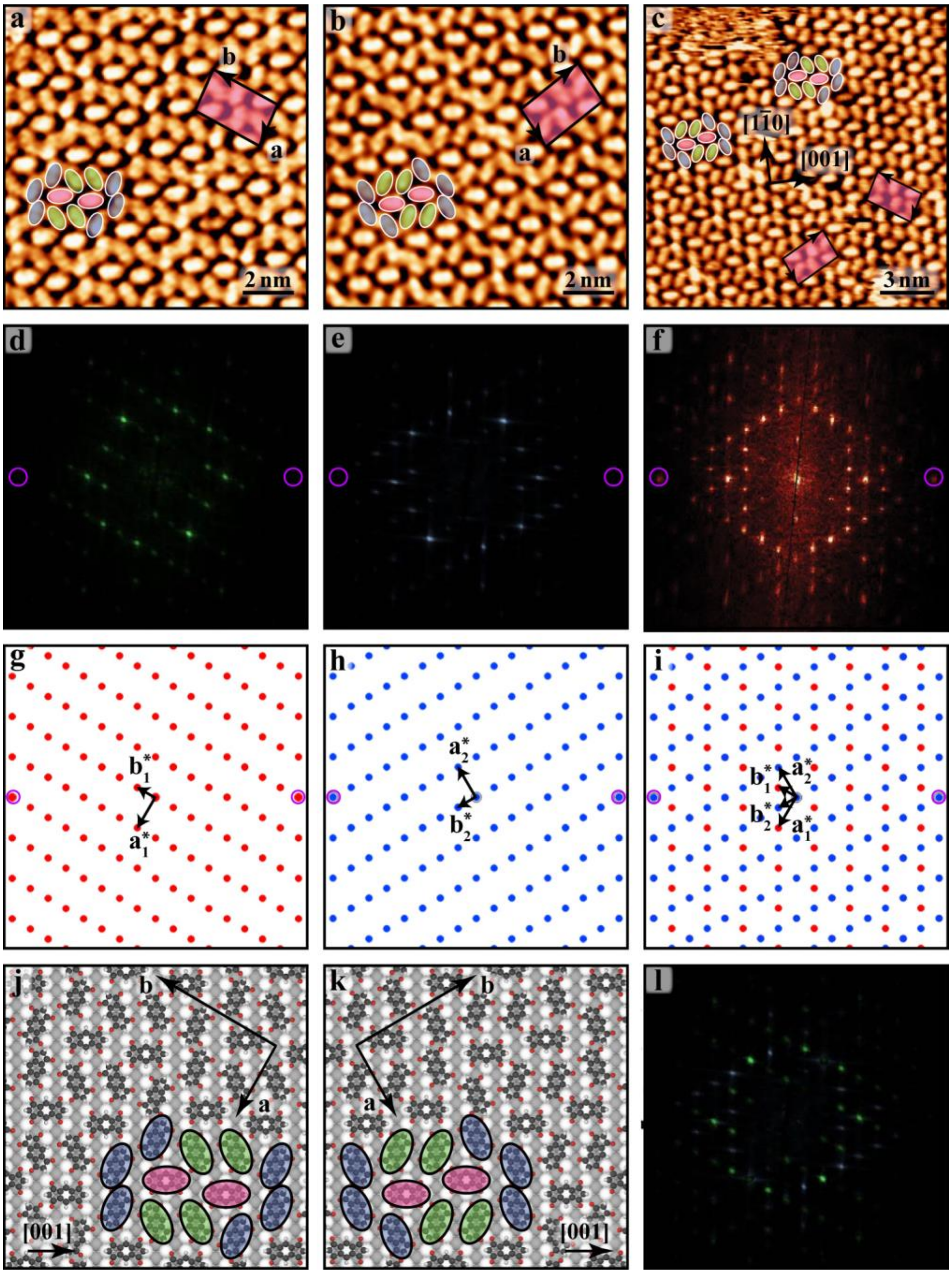

Supplementary Figure 3. Self-assembly formed by $INDO_4$ upon adsorption on Ag(110) surface held at 50 °C. (a-c) High-resolution STM images, (d-f) corresponding Fourier transforms and (g-i) simulated LEED patterns for each isolated chiral domain and for both separated by non-seamless boundary. The Ag spots are marked by the violet circles. (j,k) Tentative models of the chiral superstructures. The three different molecule orientations are marked by pink (for type A) and green/blue (for type B) oval contours. The rectangular unit cells corresponding to a (2 5; 6 -5) superstructure and lattice directions are also indicated. The superimposing of Fourier transforms corresponding to two isolated chiral domains (d,e) results in a new pattern (l) identical to that in (f).

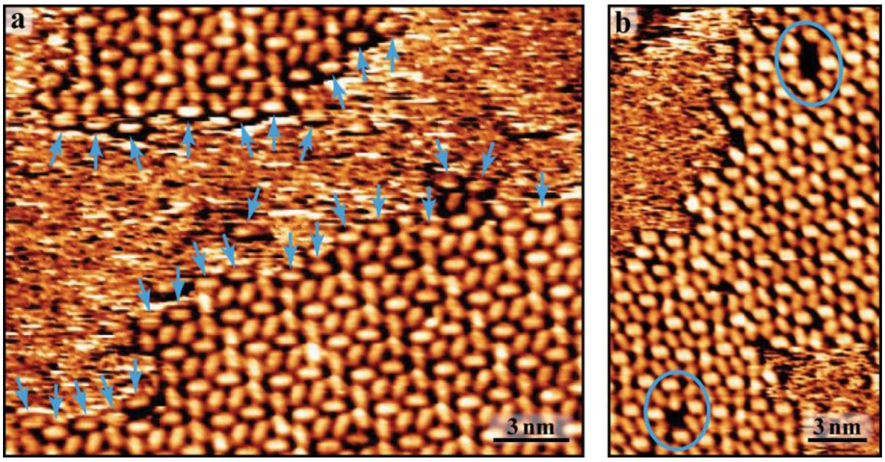

Supplementary Figure 4. Analysis of island perimeters and vacancy defects within the supramolecular self-assembly: (a) preferential termination of the molecular islands by type A molecules (pink in main text Fig. 2) indicated by blue arrows; (b) the vacancy defects in island interiors are mainly found for type B molecules (green or blue in main text Fig. 2).

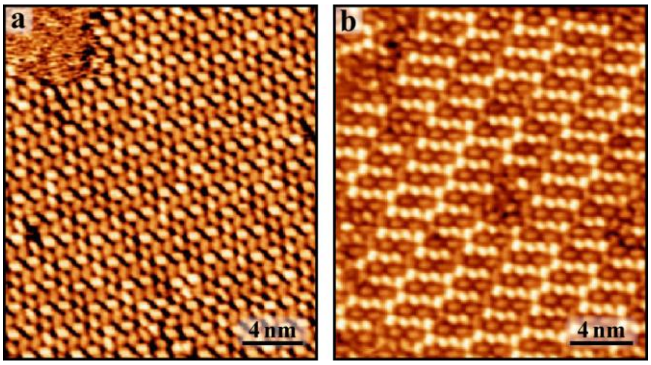

Supplementary Figure 5. STM images of the supramolecular network registered under two distinct tip imaging conditions. (a) Visualization of the backbones of $INDO_4$ type A and type B adsorption configurations as oval-shaped bright and dim protrusions, respectively, and (b) in reverse way.

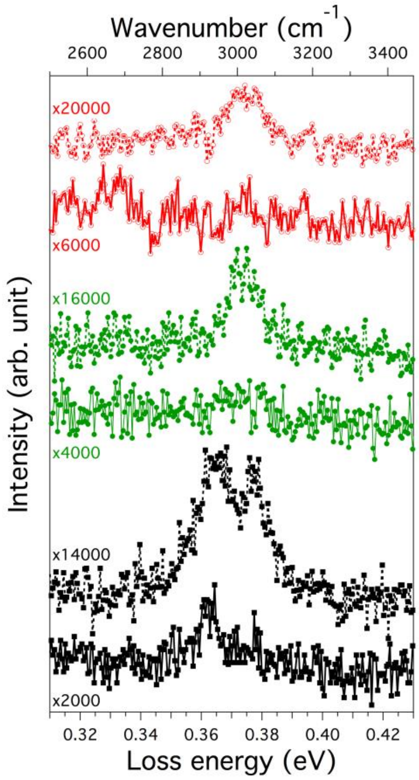

Supplementary Figure 6. Magnification of the HREELS spectra of the C–H stretching mode region taken in in- (full line with markers) and off- (dashed line with markers) specular geometry (black: thin film; green: supramolecular phase; red: polymeric phase, annealed at 380 °C). The magnification factors were chosen to normalize the background level at 4000 cm^{-1}. The disappearance of the C–H sp^3 peak at 2944 cm^{-1} suggests that the molecules underwent partial dehydrogenation due to the adsorption to the silver surface. No further evolution was observed upon annealing.

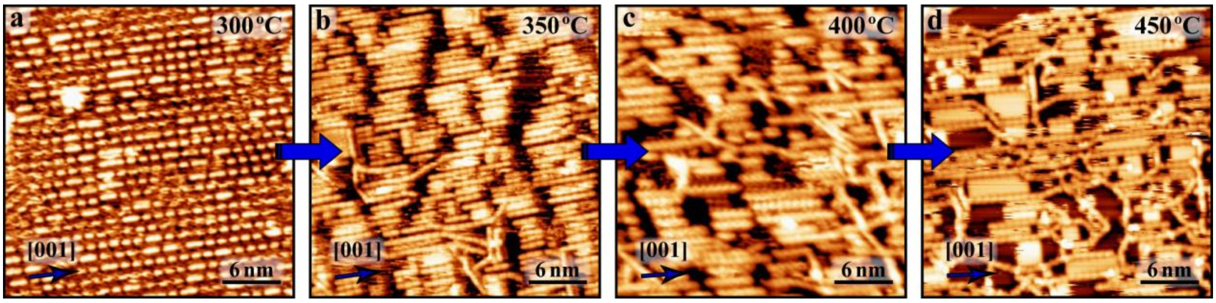

Supplementary Figure 7. STM images of the on-surface synthesis of aligned functional nanoribbons by the subsequent surface annealing or direct $INDO_4$ deposition on Ag(110) substrate held at (a) 300 °C (b) 350 °C (c) 400 °C (d) 450 °C . Close-up views of STM images in main text Fig. 4.

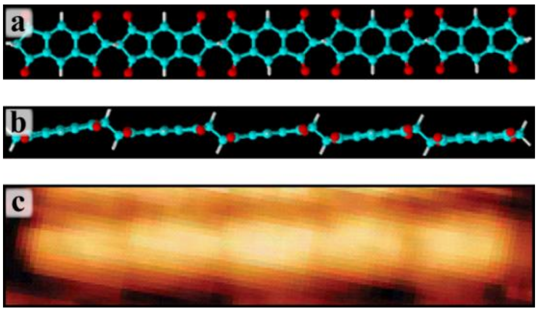

Supplementary Figure 8. Schematic representation of a pentameric nanoribbon formed by hypothetical creation of single C-C bonds (a: top view, b: side view). The chain would be non-planar, in contradiction with STM experimental data (c, extracted from main text Fig. 6c). DFT calculations on such stair-type chain provided an evaluated vibrational frequency for C*-C* stretching mode of 992 cm^{-1}, for which no peak was observed in HREELS spectra after annealing.

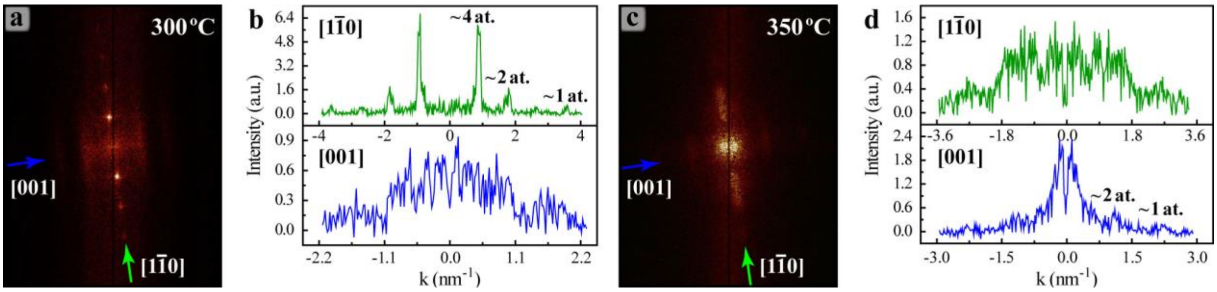

Supplementary Figure 9. Fast Fourier transforms (FFT) of STM images and line profiles along $[1\bar{1}0]$ and [001] directions of the covalent structures formed at (a, b) 300 °C (Supplementary Fig. 7a) and (c,d) 350 °C (Supplementary Fig. 7b), respectively.

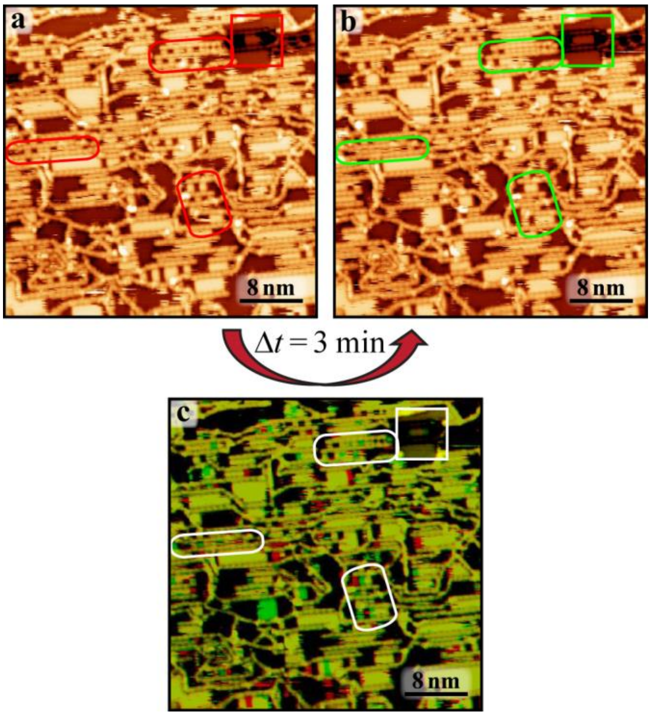

Supplementary Figure 10. Subsequent STM images of the same region of the functional nanoribbons formed by $INDO_4$ molecules upon adsorption on Ag(110) surface held at 350 °C. (a,b) Initial and final positions of the mobile molecular species indicated by red and green contours in STM images that were acquired with a time separation of 3 min, respectively. (c) Overlay of the two images modified into red and green color scale, respectively, such that stationary STM features appear approximately yellow and mobile species in green/red. Corresponding STM movie: see Supplementary Movie 1.

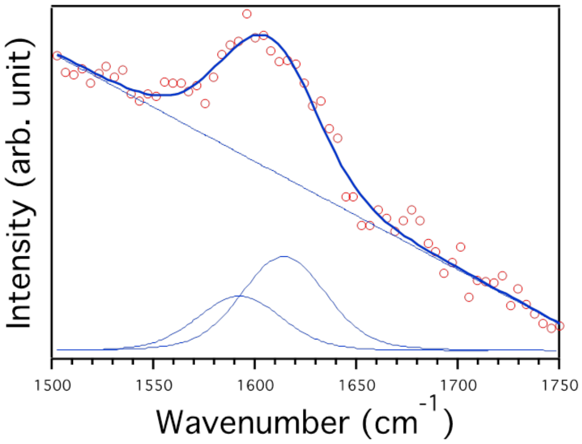

Supplementary Figure 11. Zoom-in of the HREELS data (red circle) around peak X_2 and corresponding fit (blue lines). The fitting procedure was performed in two steps. We have first fitted the elastic peak using a Voigt profile (69% Gaussian profile and 31% Lorentzian profile) with a width of 48 cm^{-1}. We then specifically used this line shape to adjust peak X_2. Its larger width of 57 cm^{-1} can accommodate two components located at 1592 cm^{-1} and 1614 cm^{-1}.

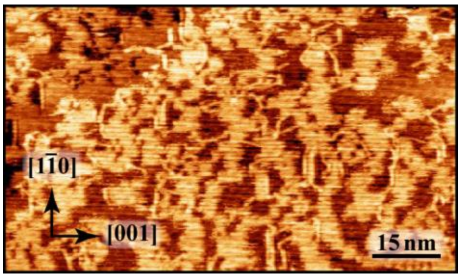

Supplementary Figure 12. STM image of the $INDO_4$/Ag(110) system after annealing at 380 °C acquired before HREELS measurement, showing a coverage of ~0.8 ML.

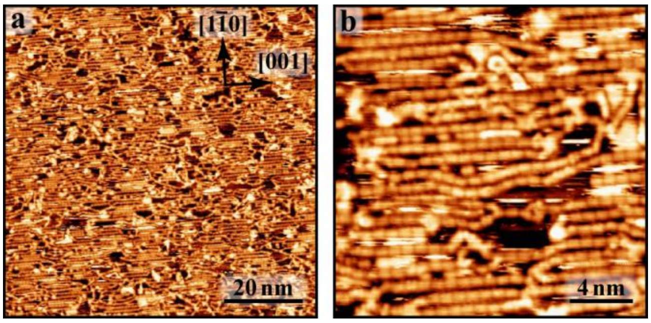

Supplementary Figure 13. STM images of $INDO_4$ nanoribbons in monolayer regime on Ag(110) after annealing up to 500 °C, showing that the covalent structures are preserved after high temperature treatment.

	B3LYP	PBE0	M06-2X	ωB97X-D	BLYP	PBE
Monomer	2.95	3.07	3.36	3.47	2.08	2.02
Dimer	1.50	1.55	1.98	1.98	0.84	0.74
Trimer	1.43	1.48	1.95	1.95	0.62	0.50
Tetramer	1.39	1.45	1.93	1.94	0.53	0.41
Pentamer	1.38	1.44	1.92	1.93	0.48	0.36

Supplementary Table 1. The evaluated absorption energy (in eV) for lowest excitation in monomer, dimer, trimer, tetramer, and pentamer forms of $INDO_4$ molecule by means of the time-dependent DFT (TD-DFT) method using different functionals. A redshift with increasing chain length was reported for all cases.
